# Molecular identification and phylogenic analysis of *Bartonella henselae *isolated from Iranian cats based on *glt*A gene

**Published:** 2016-03-15

**Authors:** Ramin Mazaheri Nezhad Fard, Seyed Milad Vahedi, Iraj Ashrafi, Faranak Alipour, Golnaz Sharafi, Hesam Akbarein, Seyed Javid Aldavood

**Affiliations:** 1 *Rastegar Central Laboratory, Faculty of Veterinary Medicine, University of Tehran, Tehran, Iran; *; 2 *Department of Internal Medicine, Faculty of Veterinary Medicine, University of Tehran, Tehran, Iran; *; 3 *Department of Microbiology and Immunology, Faculty of Veterinary Medicine, University of Tehran, Tehran, Iran; *; 4 *Department of Food Hygiene, Faculty of Veterinary Medicine, University of Tehran, Tehran, Iran.*

**Keywords:** *Bartonella henselae*, Cat, Iran, Zoonosis

## Abstract

One of the most important species of the *Bartonella* genus is *B. henselae* that causes a zoonotic infection, cat scratch disease (CSD). The main source of the bacteria is cat and the carrier is *Ctenocephalides felis *flea. One hundred and forty nail and saliva samples were collected from 70 domestic cats. Positive samples for *B. henselae* were characterized by polymerase chain reaction (PCR) and sequencing. Sequences of *glt*A gene were trimmed using BioEdit software and then compared with the sequences of the same gene from *B. henselae* isolated from cats and humans in GenBank database. Phylogenic tree was constructed using CLC Sequence Viewer software and unweighted pair group method with arithmetic mean (UPGMA) method. Molecular assessments showed that five samples out of 70 nail samples (7.14%) and one sample out of 70 saliva samples (1.42%) were genetically positive for *B. henselae*. At least an 87.00% similarity was seen between the gene sequences from the current study and the reference sequences from the GenBank database. Phylogenic analysis has shown that strains isolated in this study were grouped in a different haplo group, compared to other strains. Among the Asian countries, the prevalence of the bacteria in Iran was close to that in Japan and Turkey. In conclusion, findings of this study showed the prevalence of *B. henselae *in Iranian cats which is important due to its public health issues, especially for the immunocompromised pet owners.

## Introduction

*Bartonella* genus contains at least 20 species and subspecies of small, gram-negative, polymorphic, fastidious and hemotropic bacteria that infect humans and a variety of mammals.^1^^,^^2^ The most important species of this genus is *B. henselae* that causes a zoonotic infection, cat scratch disease (CSD). The major source of bacteria is cat and the carrier is *Ctenocephalides felis *flea. Furthermore, the bacteria has been identified in *Ixodes ricinus* ticks using polymerase chain reaction (PCR).^3^ The infection can be transferred to cats and humans by the inoculation of flea feces into the skin or mucosa. There is limited information on the role of cat bite in transfer of infection.^4^ Although most cats are normally infected with the bacteria, clinical symptoms such as fever, lymphadenopathy, endocarditis, myocarditis, gingivitis, stomatitis and uveitis have been reported in some cats.^5^ Infected cats can be remained severely bacteremic for couple of months and must be considered as potential sources of disease for cats and humans. Humans can be infected indirectly by the scratch or bite of cats, or directly by infected fleas. The bacteria causes erythematous papules in biting area and enlargement of lymph nodes in immuno-competent patients (bacillary angiomatosis) and hepatic peliosis in immunocompromised patients.^3^ Most patients with CSD have been infected by the scratch or bite of kittens or cats (usually older than 12 months).^6^ Laboratory diagnosis includes serological and molecular methods, especially PCR. Examples of commonly used samples are tissue, blood and secretions collected from lesions.^2^

*Bartonella henselae *can be divided genetically into two strains of Huston I and Marseille II.^3^ Many phylogenic studies have been carried out on these strains and have shown a high genetic diversity of the bacteria, especially in Asian isolates.^7^^,^^8^

The aim of the present study was to detect *B. henselae* in cats using molecular techniques and to phylogenetically analysis of these isolates. This is the first phylogenetic study on *B. henselae *isolated from Iranian cats.

## Materials and Methods

**Sample collection. **One hundred and forty samples (70 nail and 70 saliva samples) from 70 domestic cats (50.00% male and 50.00% female) with an average age of 13.50 months were collected in the Small Animal Teaching Hospital, University of Tehran (Tehran, Iran) from January to April 2012. Case information, including age, sex, breed, food, vaccination and symptoms, were recorded in questionnaire forms. Samples were stored at -20 ˚C until use in Rastegar Central Laboratory, University of Tehran.

**Polymerase chain reaction.** Genomic DNA extraction was carried out according to the manual instruction of the extraction kit (Bioneer, Seoul, South Korea). *Bartonella *positive samples were identified by PCR using genus-specific primers CAT1: 5'-GATTCAATTGGTTTGAAGGAGGCT-3 and CAT2: 5'-TCACATCACCAGGACGTATTC-3' amplifying a 414-bp fragment of the *htr*A gene.^9^ *Bartonella henselae *were identified using species-specific primers BartogltA-Forward: 5'-TTCCGYCTTATGGGTTTTGG-3' and Barto-henselae: 5'-CATTTCTGTTGGAAATCCTAG-3' amplifying a 246-bp fragment of the citrate synthase gene (*glt*A).^10^ The PCR reaction was prepared in a final volume of 25 μL as follows: 1X buffer, 1 mM MgCl_2_, 0.08 mM of each dNTP, 0.40 pmol of each primer, 1.00 IU of the polymerase enzyme and 100 pg to 100 ng of the DNA as template. Sufficient sterile distilled water was added to the reaction to make the final volume. The PCR thermal cycling was optimized as follows. Initial denaturation was carried out at 95 ˚C for 5 min then 35 cycles were programmed, each cycle contained denaturation at 95 ˚C for 30 sec, annealing at 57 ˚C (*htr*A) or 52 ˚C (*glt*A) for 30 sec and extension at 72 ˚C for 1 min. Final extension was performed at 72 ˚C for 7 min. A reference strain of *B. henselae *(ATCC 49793) was used as positive control. The PCR products were electrophoresed on 1.00% agarose gels.

**Sequencing. **Products of the *glt*A PCR were sequenced by Sanger method (Bioneer, Seoul, South Korea). Sequences were trimmed using BioEdit software (Version 7.2.0; An Abbott Inc., California, USA) and then were compared to cat and human sequences from GenBank database and the phylogenetic tree was constructed using CLC sequence viewer software (Version 6; CLC bio Inc., Massachusetts, USA) and unweighted pair group method with arithmetic mean (UPGMA) method.

**Statistical analysis. **Statistical analysis was performed using SPSS (Version 16; SPSS Inc., Chicago, USA) and Chi-square and Fisher tests. Confidence interval was calculated 95% and results were reported with a minimum significance of *p *≤ 0.05. The risk factor variables considered in this study included age, sex, breed and vaccination record.

## Results

**Polymerase chain reaction. **Molecular analysis showed that five samples out of 70 nail samples (7.14%) and one sample out of 70 saliva samples (1.42%) were genetically positive for *B. henselae*. Out of five positive nail samples, three were isolated from male (4.28%) and two from female (2.85%) cats. A positive saliva sample was isolated from a female cat. No significant difference was seen between the positive samples and any of the risk factor variables (*p *> 0.05).

**Sequencing. **Sequences from the six *glt*A positive isolates were deposited in GenBank database (Accession Nos. KF680530, KF680531, KF680532, KF680533, KF680534, and KF680535). At least 87.00% similarity was seen between the gene sequences in this study and other sequences from the GenBank database. Comparison between sequences of this study and Genbank sequences demonstrated 99.00% similarity with Houston I strain (accession No. BX897699; max query coverage 99.00%) and Marseille II strain (accession Nos. JN646663, JN646662, JN646661, JN646660; max query coverage 98.00%).

**Fig. 1:**
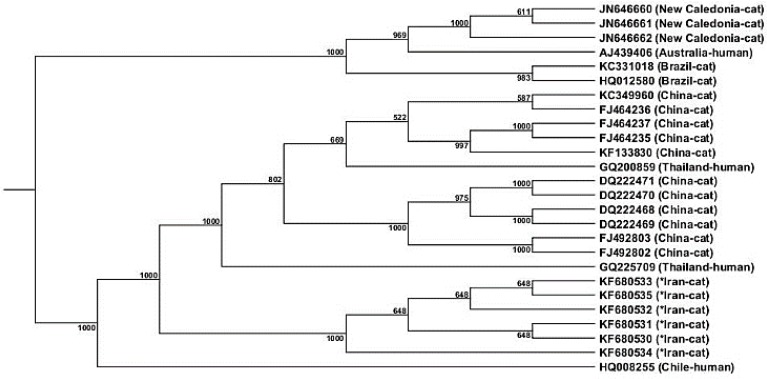
Phylogeny of *B. henselae* isolates (isolates from this study are shown with asterisks) based on *glt*A gene sequence. Bootstrap resampling was calculated 1000

**Phylogenic analysis. **Phylogenic analysis demonstrated that strains isolated in this study were grouped in a different haplogroup compared to other strains (Fig. 1). GenBank sequence isolation sources and origins used for the comparison in the current study were as follow: Cat isolates, New Caledonia (JN646660, JN646661, JN646662), Brazil (KC331018, HQ012580) and China (FJ464235, FJ464236, FJ464237, KF133830, DQ222469, DQ222471, DQ222468, DQ222470, FJ492802, FJ492803). Human isolates: Australia (AJ439406), China (KC349960) Thailand (GQ200859, GQ225709) and Chile (HQ008255).

## Discussion

Cats are the major host of *B. henselae*.^11^ Since the infection can be transferred to humans by the scratch or bite of pet cats, the public monitoring of the infection prevalence in cats is of high importance.^1^ Although no gold standard test is available to detect this micro-organism, PCR can be setup to detect *B. henselae* in biological samples as a rapid, specific and sensitive technique.^3^^,^^12^^-^^14^ Previous studies demonstrated 4.00% and 10.90% prevalence rates of the infection in domestic cats in Iran.^15^^,^^16^ However, two various methods have been used in those studies (PCR and serology, respectively). In the current study, a *B. henselae *strain was isolated from the cat nail for the first time in Iran. Although findings of the *B. henselae *prevalence in the current study are relatively close to those from the previous studies in Iran, use of various methods and sample sources prevents a statistically accurate comparison.

Distribution of the CSD is variable in Asian countries. Studies in Jordan, Thailand, Philippines, Indonesia, Singapore, Turkey, Japan and South Korea used different methods (e.g. serology and molecular biology) and samples (e.g. blood, saliva and nails) to assess the prevalence of the infection. However, the prevalence rate of infection in these countries varies from 9.10% in Japan to 62.60% in Philippines.^17^^-^^24^ In the present study, the prevalence of the bacteria was relatively low, compared to Philippines (serology, 62.60%), Indonesia (serology, 54.00%), Singapore (serology, 47.50%), Korea (PCR of saliva, 43.50%, nails 29.50%), Thailand (PCR of blood, 22.88%), but close to Japan (PCR of blood, 9.10%) and Turkey (serology, 9.40%). Further studies with similar sampling methods are required to precisely assess the prevalence of the infection. However, a high similarity between sequences has been found by Birtles and Raoult carried out exclusively on *glt*A gene sequences of various *Bartonella *species and demonstrated an excellent 99.80 to 100% similarity.^25^ According to Guy *et al*., since less than 1.00% genomic variety exists between various strains of *B. henselae*, a high genomic similarity is expected.^26^ Previous studies have shown that Houston I strain is predominant in Asia.^19^^,^^27^ In the current study, similarity between the isolates and Houston I strain was higher than that between the isolates and Marseille II strain. However, this needs to be checked by more specific primers (strain-specific) for each isolate.

In conclusion, this study showed that *B. henselae *was spread in Iranian cats which is important due to its public health issues, especially for the immuno-compromised pet owners. However, the low rate of prevalence is not concerning. The partial similarity found between the isolates in Iran and those in China and Thailand shows a potential danger of transferred infection between these countries.
